# Multivariate analysis of the exact effects of scalar position and insertion angle on speech discrimination

**DOI:** 10.3389/fneur.2025.1569100

**Published:** 2025-06-04

**Authors:** Rainer L. Beck, Antje Aschendorff, Susan Arndt, Manuel C. Ketterer

**Affiliations:** Department of Otorhinolaryngology, Medical Center—University of Freiburg, Faculty of Medicine, University of Freiburg, Freiburg, Germany

**Keywords:** cochlear implant, scalar position, scala vestibuli, speech perception, perimodiolar array, scala tympani, cochlear coverage

## Abstract

**Objective:**

Several studies examined the influence of cochlear morphology on scalar position of the electrode array and rate of dislocation. Furthermore, researchers described better speech discrimination for patients with electrode arrays positioned in scala tympani but in small study cohorts. The aim of this study is to examine the exact impact of scalar position, dislocation and angular insertion depth on postoperative speech perception.

**Design:**

We identified the patients (*n* = 531) implanted between 2003 and 2018 with a Contour Advance electrode array (Cochlear^TM^) inserted via cochleostomy by a retrospective review of the Cochlear Implant Database and analyzed the postoperative imaging by cone beam computed tomography and the audiological protocol via a multivariate nonparametric analysis.

**Results:**

The multivariate nonparametric analysis of this study shows, that the dislocation of the electrode array and the insertion angle leads to no significant different postoperative speech discrimination results. Nevertheless, we could calculate a statistically significant amount of reduced speech recognition for monosyllables for primary scala tympani vs. scala vestibuli insertions (7.6%).

**Conclusion:**

This study, based on one of the largest study cohorts published to date, demonstrates reduced speech recognition for scala vestibuli insertions compared to scala tympani insertions. Insertion into the scala vestibuli results in a 7.6% decrease in speech discrimination for monosyllables.

## Introduction

Speech discrimination outcomes after cochlear implantation (CI) vary widely and are influenced by numerous factors, many of which remain incompletely understood. Factors such as etiology (1), the duration of hearing loss, and prior use of hearing aids (2) significantly contribute to these variations. While some factors lie beyond the control of the implanting surgeon, the question of whether specific implantation techniques or electrode array positioning can improve audiological outcomes remains pertinent.

Aschendorff et al. (3) demonstrated the benefit of electrode arrays positioned in the scala tympani in a study involving 43 patients, findings that were subsequently confirmed by Finley et al. (4) and Holden et al. (5). To support future advancements, minimizing cochlear trauma is crucial to prevent avoidable damage to the modiolus and the spiral ganglion (6). Early studies, such as those by Escudé et al. (7) and Eshraghi et al. (8), explored the impact of cochlear morphology on damage caused by dislocated electrode arrays. Escudé et al. (7) introduced the measurement of cochlear diameter A, representing the largest distance from the round window, and the perpendicular diameter B. Ketterer et al. (9) found that a smaller cochlear size increases the risk of scala vestibuli insertions and dislocations. Jwair et al. (10) described that in 32 inserted temporal bones, scalar dislocation occurred significantly more often with perimodiolar electrode arrays compared to lateral wall electrode arrays. Furthermore, they published a meta-analysis of 33 studies, reporting significantly more dislocations and tip fold-overs in perimodiolar electrode arrays compared to straight ones, with an occurrence rate of 43% vs. 7% (11). Additionally, a retrospective study of 140 patients with residual hearing further supported these findings (12).

Complementing these findings, Ketterer et al. (13) reported that increasing cochlear coverage does not necessarily result in significantly better speech perception outcomes. However, quantifying the extent of poorer speech discrimination associated with scala vestibuli insertions compared to correctly positioned scala tympani insertions remains challenging and statistically complex. The aim of this study is to investigate the influence of scalar position and dislocation on speech perception outcomes for one of the most inserted electrode arrays worldwide.

## Materials and methods

### Patient selection

We included all adult patients implanted between 2003 and 2018 with a Contour Advance (CA) electrode array by Cochlear™. Even though, there are other electrode arrays available the Contour Advance electrode array is still one of the most inserted and therefore of interest to examine. Patients were identified through a retrospective review of the CI Database. All candidates had profound bilateral hearing loss with insufficient speech discrimination while using hearing aids and were 18 years or older at the time of surgery. Surgery was performed using a facial recess approach following mastoidectomy by experienced surgeons. The CA electrode array was inserted via cochleostomy in all cases. As described by Rebscher et al. (14) and Souter et al. (15) the CA is specifically designed for the cochleostomy approach due to higher cochlear trauma if inserted via the round window. All CA electrode arrays were advanced to the first or second silicone ring, and occasionally to the third, in accordance with the protocol described by Fraysse et al. (16).

Patients included in the study were those undergoing first-time implantation with regular cochlear anatomy. We excluded patients with signs of obliterative diseases (e.g., meningitis, labyrinthitis, or otosclerosis) or signs of malformations of the labyrinth (e.g., Mondini malformation and X-linked conditions) or those who had previously undergone stapedectomy, stapedotomy, or cholesteatoma surgery, and all patients under the age of 18 at the time of implantation.

### Imaging

Postoperative imaging of the cochlea and electrode array was obtained using a DynaCT-equipped Axium Artis dTA angiography unit (Siemens Co., Erlangen, Germany) with a digital flat-panel detector. This imaging method provides multiplanar reconstructions with smaller electrode artifacts compared to conventional computed tomography and offers a voxel size of 0.1 mm.

Three specialists—two highly experienced ENT surgeons and a radiologist—independently evaluated cochlear anatomy and the scalar position of the electrode array. This technique has been validated in temporal bone studies through comparison with histological findings and has been applied in human insertion studies (9, 13, 17–20). Cochlear dimensions (7) and the insertion angle (from cochleostomy to electrode array tip) were measured separately, as described by Ketterer et al. (9, 13, 20), Beck et al. (21) and Everad et al. (22).

### Speech audiometry

Open-set speech discrimination was evaluated in a sound-treated chamber under standard clinical conditions using Freiburg monosyllables for each ear separately, with sufficient masking. Testing was conducted at a presentation volume of 65 dB SPL from 2014 onward and 70 dB SPL from 2003 to 2013 under quiet conditions, as the in-house testing protocol was revised in 2014. Speech discrimination was scored as the percentage of correctly identified words. Audiological measurements followed the standard rehabilitation schedule.

### Statistics

The resulting effects are usually small and easily disturbed by heterogeneous groups. The same problem is present if longitudinal measurements are coalesced in discrete time intervals. We therefore chose mixed-effect models as our strategy to eliminate the need for arbitrary decisions in regard to the forming of different cohorts. After fitting a nonlinear regression (asymptotic regression) through origin characterized by asymptotic value—Asym and the logarithm of the rate constant—lrc to the audiological data of every patient individually using Gnu R in conjunction with the package NLME (23), several mixed-effect models were constructed, including different covariates. The “fitness” of a distinct model, i.e., how well it represents the underlying data, can be judged by comparing entropy. Difference in fit is judged by ANOVA. In models that provided a significant improvement of fit, the significance and effect of the covariates were analyzed by ANOVA.

## Ethics committee

We performed this retrospective study at the department of Otorhinolaryngology at the University Medical Center of Freiburg. This study was approved by the Hospital's Ethics Committee according to the declaration of Helsinki (Washington, 2002) (Number of ethic committee approval: 406/19) and registered on German Clinical Trials Register (www.drks.de/DRKS00019807).

## Results

### Subjects

We identified and included 531 ears that received a CI with a CA electrode array during the specified years. Age at surgery ranged from 18.2 to 93 years (mean 48.8 years). After splitting the patients according to the position of the electrode array, no significant difference could be found regarding the age at implantation (ANOVA, *p* > 0.05).

The time between initial activation of the implant and last measurement ranged from 26 to 3,219 days (mean 1,290 days for monosyllables).

### Imaging

In some cases imaging data could not be sufficiently acquired due to missing criteria such as inadequate image resolution, not visualized round window or cochleostomy, or the inability to clearly distinguish scalar positioning in the reconstruction. These cases were excluded from the study. Cochlear alterations due to fibrosis (e.g. meningitis, otosclerosis, and labyrinthitis) were most commonly observed alongside malformations of the labyrinth (e.g. Mondini malformation, X-linked conditions). Measurements of the insertion angle and cochlear size were conducted later and required a complete three-dimensional dataset. However, some reconstructions for scalar positioning were performed earlier, and the raw data was no longer available.

**Figure 1:**
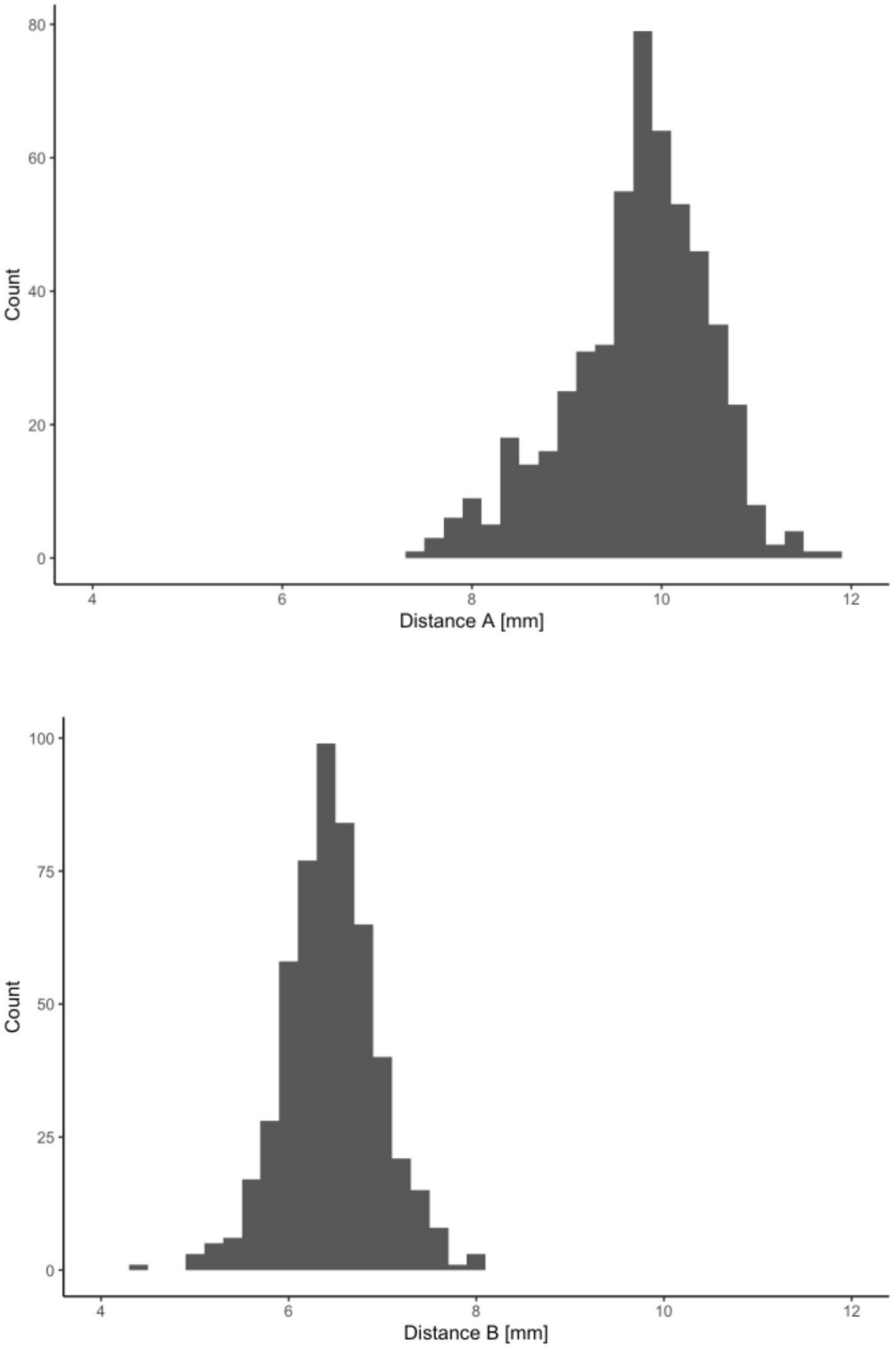
Distribution of cochlear diameter A measured as the largest distance from the round window through the modiolus, and the perpendicular diameter B as established by Escudé et al. (7).

Retrospectively, some imaging had been conducted at another clinic using computed tomography with a substantially larger voxel size, or intraoperative imaging techniques were employed, often due to malformations. As a result, 531 ears were available for precise measurements and evaluations. Cochlear morphology was assessed using diameters A and B, as described by Escudé et al. (7). Figure 1 illustrates the distribution of cochlear sizes for both diameters and shows comparable results to previously published studies (7, 9, 24) with diameter A in mean 0.8 mm +/– 0.75 (max: 11.9 mm; min: 7.4 mm) and diameter B with mean of 6.5 +/– 0.5 mm (max: 8.1 mm; min: 4.5 mm). This confirms the plausibility check of the measurements.

**Figure 2:**
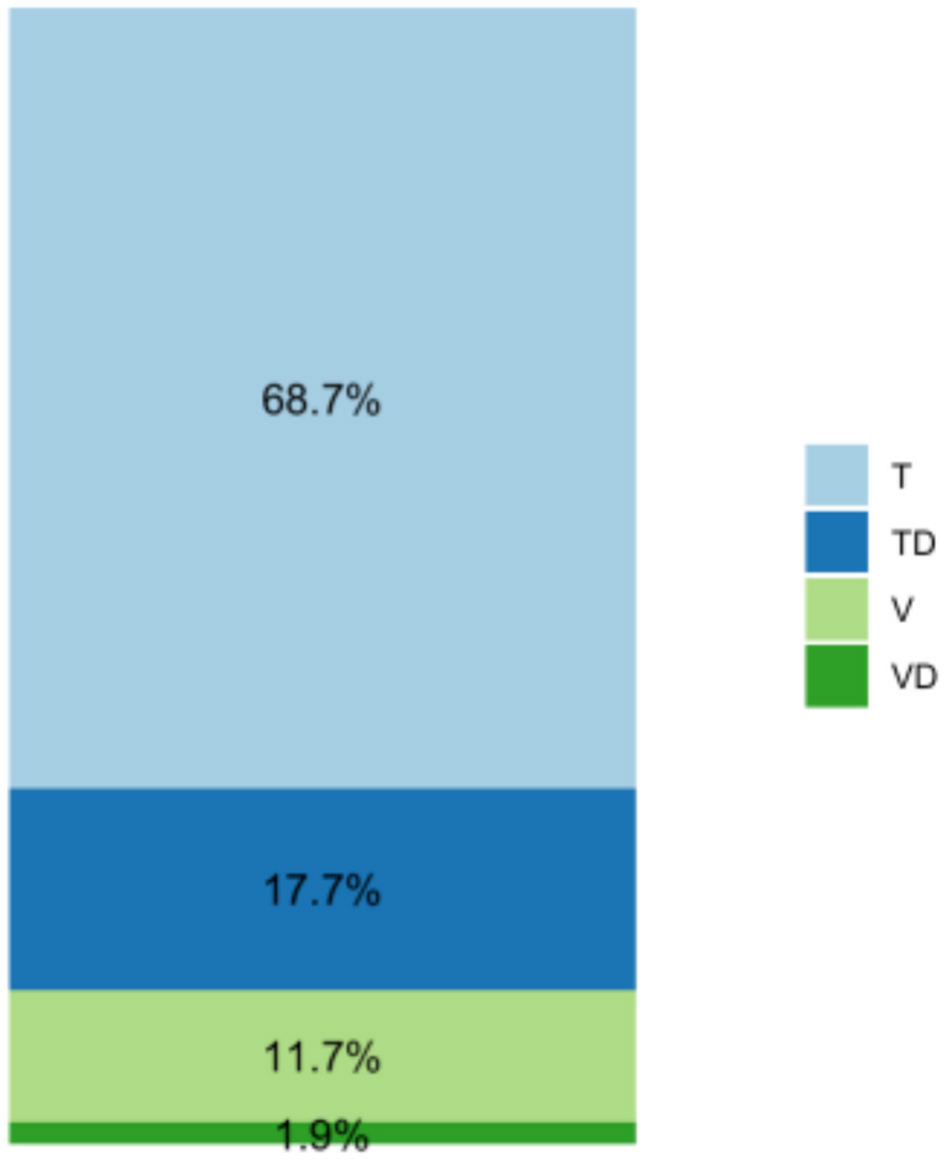
Distribution of the scalar position for the included Contour Advance (CA) electrode arrays (*n* = 531), (Cochlear^TM^). (T = scala tympani, TD = dislocated out of scala tympani, V = scala vestibuli, VD = dislocated out of scala vestibuli).

**Figure 3:**
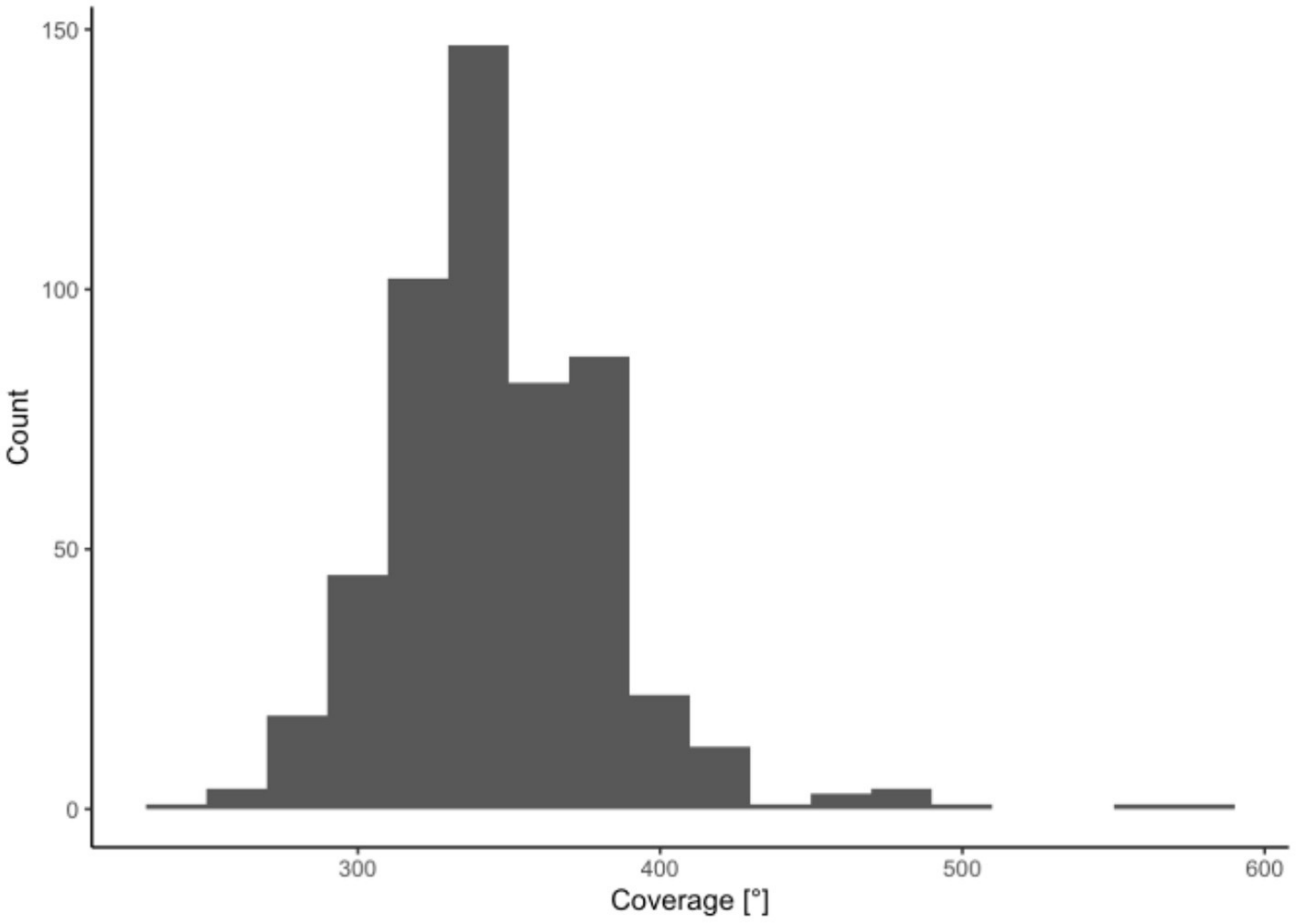
Distribution of cochlear coverage (in°) for the included electrode array (*n* = 531).

The scalar position of the electrode array was determined in all these cases. As shown in Figure 2, a complete scala tympani insertion was most common (68.7%), followed by dislocation out of scala tympani (17.7%), scala vestibuli (11.7%), and dislocation out of scala vestibuli (1.9%). One electrode array was initially inserted into the scala media and was therefore excluded from further analysis. Angular insertion depth demonstrated a mean of 347° +/−38° (min: 241°, max: 585°) (see Figure 3).

### Effects of scalar position and insertion angle

Audiometric data was retrospectively analyzed from our database. Only long-term stable audiometric results with more than three recurring stable measurements (*n* = 456) were included. Audiometric testing was conducted over an average duration after first fitting of 3.7 years (minimum: 0.7, maximum: 8.8 years).

**Figure 4:**
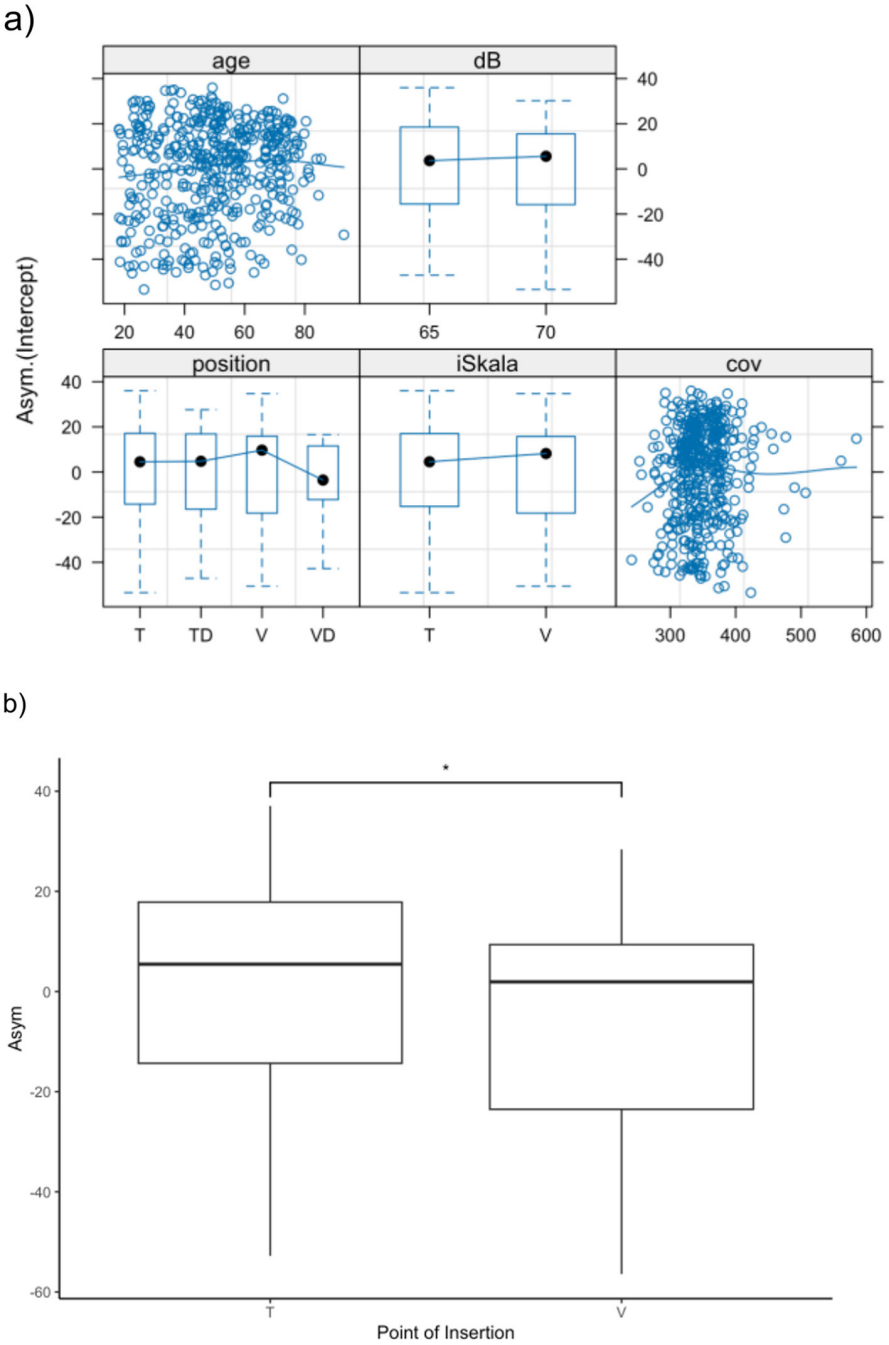
**(a)** Presentation of the mixed effect model with presentation level as a known factor and modeling the asymptotic value (Asym) for speech discrimination in monosyllables. While we could not find significant influences for age **(a)**, dislocation and coverage, primary scalar insertion (T vs. V) demonstrates significant influence **(b)**. *p < 0.05.

To account for the fact that measurements were performed at 65 dB SPL from 2014 to 2018 and at 70 dB SPL from 2003 to 2013, we constructed a mixed-effects model and included the presentation level as a known factor. The best fit for the population was achieved by modeling the asymptotic value (Asym) for speech discrimination. A significant effect on Asym was observed for the scalar position when comparing scala tympani and scala vestibuli insertion of the electrode array. Specifically, Asym decreased by 7.6 percentage points (*p* = 0.02) (see Figure 4).

No significant effect was found for either the insertion angle or the dislocation of the electrode array into another scala. To evaluate the effect of scalar dislocation and insertion angle in a more homogeneous population, electrode arrays primarily inserted into scala tympani were analyzed as a subgroup. Similarly, no significant effect of scalar dislocation or insertion angle was observed in this subgroup.

## Discussion

### Impact of scalar position and dislocation on speech discrimination

Scalar position, primary insertion in scala tympani instead of scala vestibuli, is understood as one of the markers for quality of surgery. This study confirms that primary scala tympani insertions are favorable in CI surgery and correlating to an increased postoperative speech perception by 7.6 % in monosyllables compared to scala vestibuli inserted patients. Aschendorff et al. (3) first examined scalar position via rotational tomography for patients inserted with a Contour (*n* = 21) vs. a Contour Advance (*n* = 22) electrode array and described significantly higher speech discrimination results for primary insertion in scala tympani compared to scala vestibuli. Skinner et al. (25) examined 15 patients implanted with an Advanced Bionics device postoperatively examined via CT and described a significantly negative correlation between the number of electrodes located in the scala vestibuli and the monosyllabic postoperative outcome. Our results confirm the results of the previous work with increased statistical rigor in one of the largest study cohorts published so far examining one electrode array exclusively.

Previous comparable literature with a sufficiently large sample size and, consequently, meaningful statistical power is scarce. Gu et al. (26) included only 12 scala vestibuli insertions vs. 21 scala tympani insertions, of which 9 out of 12 scala vestibuli insertions were at least partially ossified. Therefore, their conclusion that scala vestibuli insertion does not lead to detrimental hearing outcomes is highly questionable. Das et al. (27) reported that scala vestibuli insertions result in auditory outcomes comparable to scala tympani insertions. However, their review analyzed 17 studies with a total of only 72 patients and included bacterial meningitis as the leading cause of hearing loss (28%). This condition was excluded from our study due to the well-established fact that both auditory and cognitive rehabilitation following meningitis differ significantly and are often associated with cochlear ossification. In contrast, James et al. (28) demonstrated in a large cohort of 118 subjects that speech perception scores were negatively correlated with the proportion of the active electrode array located in the scala vestibuli. We confirm this finding in our study with a cohort of 456 patients. Holden et al. (5) (*n* = 114) performed a nonparametric multivariate analysis of variance (NPMANOVA) for different variables e.g., scalar position. Different electrode arrays and different manufacturers were included. Addressing part of the statistical dilemma using retrospective data, the authors divided the study subjects in different subgroups according to their CNC word score still. Nevertheless, a positive impact of primary insertion into scala tympani could be found. To improve rigor, we concentrated on one manufacturer and one electrode array only.

Holden et al. (5) and Finley et al. (4) suggested that scala vestibuli insertions may cause pitch confusion and reduced speech recognition due to cross-turn stimulation. Additionally, cochlear trauma has been proposed as a main reason for poorer outcomes. Animal studies (29) showed that such trauma reduces spiral ganglion cells, though no clear link between ganglion cell survival and outcomes was found (30). Ganglion cell counts cannot be assessed in patients. Therefore, the direct effect of residual ganglion cell activity on scala vestibuli insertion remains speculative. Nevertheless, some studies have described an indirect way for clinicians to assess the function and condition of the auditory nerve through electrically evoked compound action potentials, thereby estimating the amount of viable spiral ganglion cells in guinea pigs (31, 32).

Finley et al. (4) calculated via a linear regression analysis for 14 patients with dislocated electrode arrays that scalar position and total number of electrodes within the scala vestibuli accounted for 83% of the variance in monosyllabic word scores. It has to be noted, that the study included three different electrode arrays and the audiological data was acquired 4–36 months following implantation. In our opinion, the heterogeneity of the study cohort would not allow for this kind of conclusion.

In addition, Boyer et al. (33) showed a relative narrow window of dislocation for the CA between 170 and 190 degrees (*n* = 39) that makes a linear correlation unlikely for this kind of precurved electrode array. Ketterer et al. (13) could confirm the electrode array design specific point of dislocation at around 180° for the CA electrode array. The findings of our study show no significant effect of dislocation on postoperative speech perception. This may be due to the relative importance of stimulation of the basal turn of the cochlea compared to the apical regions about speech discrimination or to a relative small effect size that could not be detected by our study cohort. Nevertheless, it is important to highlight that scala vestibuli insertion is more related to surgical factors, particularly the location of the cochleostomy, whereas scalar dislocation is associated with insertion dynamics (34). In contrast to round window insertions, a cochleostomy disrupts the integrity of the cochlear inner ear, potentially leading to inflammation and fibrosis, which may result in a reduction of viable inner hair cells and spiral ganglion cells. Therefore, we exclusively evaluated one electrode array (CA) and included only cochleostomy approaches.

### Impact of insertion angle on speech discrimination

The insertion angle measured in this study showed no significant influence on monosyllable discrimination following CI. Therefore, we conclude that cochlear coverage is not influencing postoperative speech perception for the included electrode array. Several studies published before (35, 36) described a significant positive correlation between angular insertion depth and monosyllabic results detected 1 year postoperatively. Whereas, Finley et al. (4) found a negative correlation between insertion depth and monosyllabic word results in 14 patients implanted with a device of Advanced Bionics. The aforementioned investigations (4, 35, 36) included a relatively small, inhomogeneous sample size compared to this study.

Holden et al. (5) measured 114 ears and described that angular insertion depth is negatively correlated with CNC Final Score and negatively related to their established outcome group. It has to be noted that Holden et al. (5) included two different implant systems (Cochlear and Advanced Bionics) and at least 4 different types of electrode arrays in their study. They haphazardly established outcome groups and did not calculate the influence of the insertion angle on postoperative speech discrimination for their total group of included study patients. Finley et al. (4) and Holden et al. (5) have few participants with shallow insertions and they therefore describe their measured insertion depth as negatively correlated to the postoperative speech recognition results, but included shallow insertions to their study cohort. Our research group (13) described a negative influence of increasing cochlear coverage on speech perception in their large total study cohort for the total cohort, but could not confirm it calculating it separately for each included manufacturer. These results can be confirmed again with the included electrode array CA of Cochlear^TM^ of this study. Nevertheless, the retrospective nature of the study is a limitation that must be mentioned. Furthermore, pre- and postoperative pure-tone audiometry data could be a good indicator of cochlear function preservation but were not collected in this study due to its retrospective design.

## Conclusion

This study, based on one of the largest study cohorts published to date, demonstrates the extent of reduced speech recognition for scala vestibuli vs. scala tympani insertions. An insertion into the scala vestibuli decreases monosyllabic speech recognition by 7.6%, highlighting the need for expertise and precision in cochleostomy placement. Nevertheless, scalar dislocation does not result in significantly reduced speech recognition following CI.

## Data Availability

The raw data supporting the conclusions of this article will be made available by the authors without undue reservation, subject to approval by the local ethics committee.
